# Investigation of the acute pathogenesis of spondyloarthritis/HLA-B27-associated anterior uveitis based on genome-wide association analysis and single-cell transcriptomics

**DOI:** 10.1186/s12967-024-05077-y

**Published:** 2024-03-12

**Authors:** Shuming Chen, Weidi Huang, Qiaoqian Wan, Zichun Tang, Xie Li, Fang Zeng, Shuyan Zheng, Zhuo Li, Xiao Liu

**Affiliations:** 1 Department of Ophthalmology, The Second Xiangya Hospital, Central South University, Changsha, 410011 China; 2 Hunan Clinical Research Center of Ophthalmic Disease, Changsha, 410011 Hunan China; 3 Department of Anaesthesiology, the Second Xiangya Hospital, Central South University, Changsha, 410011 China; 4 Hunan Provincial Key Laboratory of Critical Quality Attribute of Cell Therapy Products, Changsha, 410011 Hunan China

**Keywords:** Anterior uveitis, Genetics, Mendelian randomization, Single-cell transcriptome

## Abstract

**Background:**

Patients with spondyloarthritis (SpA)/HLA-B27-associated acute anterior uveitis (AAU) experience recurring acute flares, which pose significant visual and financial challenges. Despite established links between SpA and HLA-B27-associated AAU, the exact mechanism involved remains unclear, and further understanding is needed for effective prevention and treatment.

**Methods:**

To investigate the acute pathogenesis of SpA/HLA-B27-associated AAU, Mendelian randomization (MR) and single-cell transcriptomic analyses were employed. The MR incorporated publicly available protein quantitative trait locus data from previous studies, along with genome-wide association study data from public databases. Causal relationships between plasma proteins and anterior uveitis were assessed using two-sample MR. Additionally, colocalization analysis was performed using Bayesian colocalization. Single-cell transcriptome analysis utilized the anterior uveitis dataset from the Gene Expression Omnibus (GEO) database. Dimensionality reduction, clustering, transcription factor analysis, pseudotime analysis, and cell communication analysis were subsequently conducted to explore the underlying mechanisms involved.

**Results:**

Mendelian randomization analysis revealed that circulating levels of AIF1 and VARS were significantly associated with a reduced risk of developing SpA/HLA-B27-associated AAU, with AIF1 showing a robust correlation with anterior uveitis onset. Colocalization analysis supported these findings. Single-cell transcriptome analysis showed predominant AIF1 expression in myeloid cells, which was notably lower in the HLA-B27-positive group. Pseudotime analysis revealed dendritic cell terminal positions in differentiation branches, accompanied by gradual decreases in AIF1 expression. Based on cell communication analysis, CD141^+^CLEC9A^+^ classic dendritic cells (cDCs) and the APP pathway play crucial roles in cellular communication in the Spa/HLA-B27 group.

**Conclusions:**

AIF1 is essential for the pathogenesis of SpA/HLA-B27-associated AAU. Myeloid cell differentiation into DCs and decreased AIF1 levels are also pivotal in this process.

**Supplementary Information:**

The online version contains supplementary material available at 10.1186/s12967-024-05077-y.

## Introduction

Spondyloarthritis (SpA)/HLA-B27-associated acute anterior uveitis (AAU) is a subtype of anterior uveitis [1–3]. Its prevalence in young adults is characterized by easily repeatable acute monocular episodes, which can seriously endanger the patient's vision and impact their ability to work, resulting in a heavy economic burden [4]. Several studies have established strong links between SpA or HLA-B27 and AAU [1, 3, 5], but the exact pathogenesis of these correlations has not been determined.

Various hypotheses have been proposed regarding the acute pathogenesis of SpA/HLA-B27-associated AAU. The ketogenic/arthritogenic peptide hypothesis suggests that the pathogenesis of SpA or AAU is an autoimmune response mediated by cytotoxic T cells [6]. Since HLA-B27 molecules are MHC-I molecules that are natural antigen-presenting molecules, they can bind to certain specific peptides in joint or eye tissues and present them to other immune cells to activate an autoimmune response. These uveitogenic/arthritogenic peptides can be either exogenous or endogenous. The HLA-B27-derived peptide hypothesis suggests that misfolding of the HLA-B27 molecule leads to its presentation as an antigen by MHC-II-like molecules, which in turn activates CD4^+^ T cells [7]. This interpretation partially explains the role of CD4^+^ T cells in the pathogenesis of SpA/HLA-B27-associated AAU. The intestinal microbiota hypothesis suggests that changes in intestinal permeability caused by the intestinal microbiota lead to monitoring of bacterial antigens via the immune system. Some of these bacterial antigens can cause abnormal activation of the immune system through molecular mimicry, resulting in an imbalance in the homeostasis of the immune microenvironment, such as a decrease in the number of certain regulatory T cells and abnormal DC cell presentation [8]. This ultimately leads to the migration of immune cells to the eye or other organs, causing disease. All of these hypotheses suggest that alterations in the immune microenvironment are important for causing SpA/HLA-B27-associated AAU. Overall, identifying the underlying mechanisms is important for prevention and treatment of acute anterior uveitis. However, due to the complexity of the immune microenvironment, in which each alteration in the microenvironment causes a dramatic change in the entire microenvironment, many confounding factors make it difficult to experimentally identify the key steps involved.

Many research methods based on big data and multiomics have emerged to provide scientific means to elucidate the pathogenesis of complex diseases. Genome-wide association studies (GWASs) identify locus‒phenotype associations by testing hundreds of thousands to millions of genetic variants in the genomes of numerous individuals. This approach allows for simple biological interpretation of association results and identification of novel biomarkers and drug targets [9, 10]. The phenotype can be a complex disease or a simple phenotype, such as expression of a gene or the level of a plasma protein. Since plasma proteins play key roles in a variety of biological processes, such as signalling, growth, repair, and immune defence, they are reliable drug targets [11]. Therefore, many scholars have identified various genetic determinants of protein expression by performing GWASs at the level of plasma proteins, and these determinants are known as protein quantitative trait loci (pQTLs) [12], which can be utilized to study the correlation between plasma proteins and diseases [13]. GWASs as well as identification of a considerable number of loci associated with complex traits or diseases have been performed; however, most do not directly affect protein-coding regions, which greatly hampers our understanding of the molecular basis of human disease [14]. Therefore, GWASs often need to be complemented with evidence provided by basic experiments, which suffer from low throughput and time-consuming problems.

The concept of Mendelian randomization (MR) was systematically described by Davey Smith G et al. as early as 2003 [15]; it is a statistical method based on GWASs in which genetic variants are used as proxy variables to infer causal relationships. The basic idea of MR is to select genetic variants as instrumental variables to represent the exposure factor and to use the association statistics between the variant and the exposure and with the outcome to estimate the causal effect between them. Since alleles are randomly segregated and passed on to offspring at the time of parental gamete formation, this natural method of random assignment is even superior to that of randomized controlled trials. In addition, the genetic material identified at birth is virtually immune to interference from acquired social and environmental confounders, and its chronological relationship to the outcome predates the study outcome, thus avoiding reverse causal inference.

Although MR excels in inferring causality, it is unable to provide a clear explanation of the underlying mechanisms involved. In recent years, single-cell sequencing technology has become increasingly sophisticated, with increased resolution to the individual cell level, providing unparalleled accuracy for studying disease mechanisms [16, 17]. However, due to its high precision, any confounding factors during the sequencing process can dramatically affect the results. Moreover, the application of single-cell sequencing technology requires correct a priori knowledge to identify confounding factors and key disease-causing factors. Therefore, single-cell sequencing and MR analysis well complement each other to identify the initiating factors and key pathways involved in disease pathogenesis.

Accordingly, we used an MR analysis approach to analyse publicly available GWAS data to identify key plasma proteins involved in the pathogenesis of SpA/HLA-B27-associated AAU. We then performed single-cell transcriptome sequencing to investigate the specific mechanisms involved. This study provides an important theoretical basis for monitoring the pathogenesis of SpA/HLA-B27-associated AAU and for drug development.

## Methods

### Study design and ethics

**Fig. 1:**
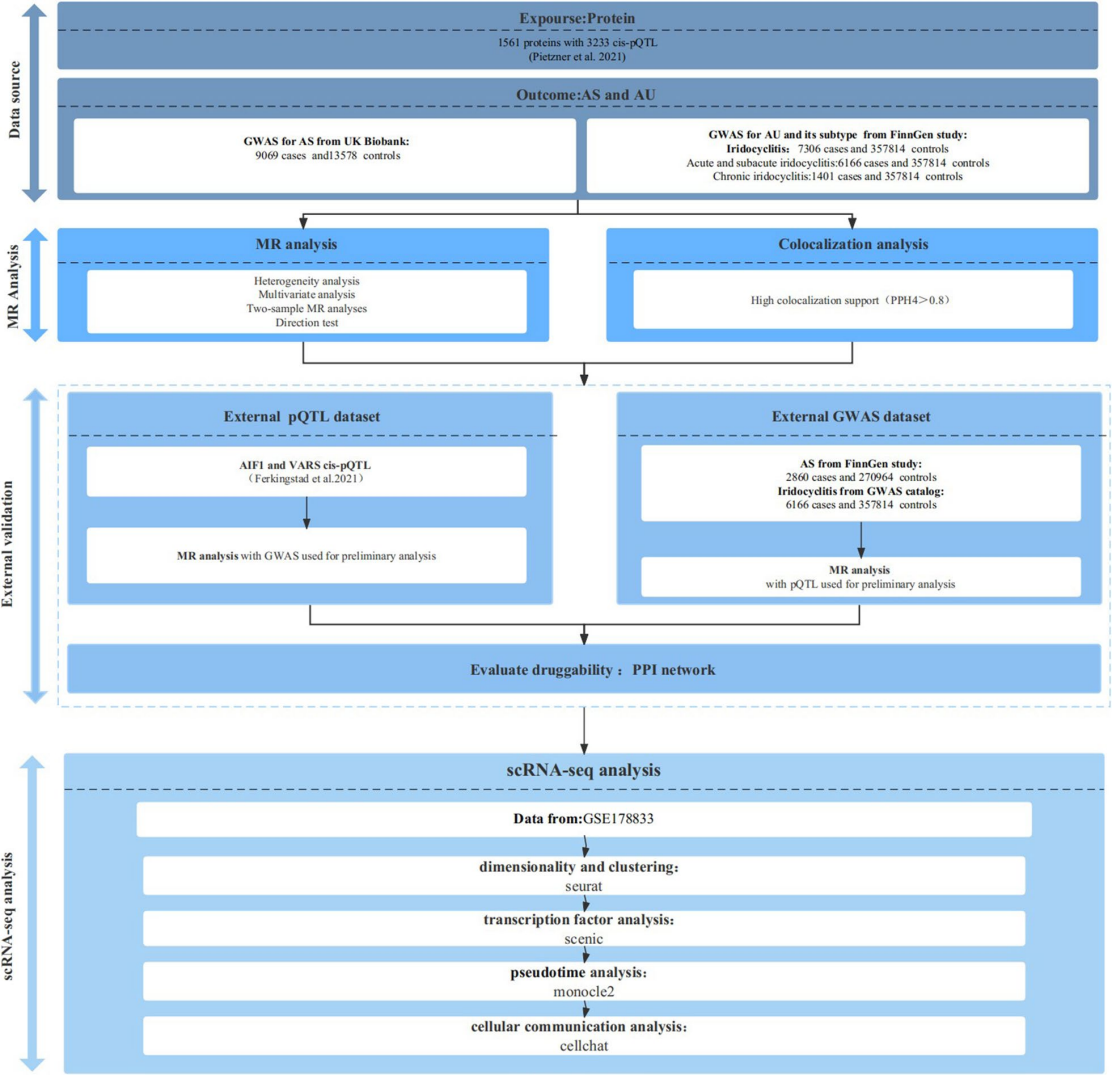
Study design. pQTLs, protein quantitative trait loci; *AS* ankylosing spondylitis; *AU* anterior uveitis, *GWAS* genome-wide association study, *MR* Mendelian randomization, *PPI* protein–protein interaction, *scRNA-seq* single-cell transcriptomics

The flowchart of the study is presented in Fig. 1. The study was divided into two parts: MR analysis and single-cell transcriptome analysis. MR analyses were conducted using GWAS summary statistics and large-scale pQTL statistics. We obtained pQTL data from published studies by Pietzner et al. [18] and Ferkingstad et al. [19] and collected GWAS statistics from UK Biobank, the FinnGen study, and GWAS Catalog. The present study utilized the single-cell transcriptome sequencing dataset GSE178833 obtained from the GEO database for analysis of AAU in HLA-B27-positive patients. As previously collected and published data were reanalyzed, no further ethical approval was needed.

### MR analysis

#### Plasma protein quantitative trait loci

The plasma pQTL data used in our preliminary analysis were obtained from a previous study by Pietzner et al. [18]. Their study measured protein targets from 10,708 participants of European ancestry using the SomaScan v4 assay and identified a total of 3,323 cis-pQTLs and 7314 trans-pQTLs. In our study, we included only proteins with cis-pQTLs that reached genome-wide significance (P < 5 × 10^–8^) in the MR analysis. For validation purposes, we used pQTL data from Ferkingstad et al. [19]. Proteomic analyses utilizing the SomaScan v4 assay were conducted on 35,559 individuals of European descent.

#### GWAS summary statistics

In this study, ankylosing spondylitis (AS) was utilized as a surrogate phenotype for HLA-B27-positive spondyloarthritis, and iridocyclitis was employed as a surrogate phenotype for anterior uveitis. Preliminary analysis involved GWAS data sourced from UK Biobank for AS and from the FinnGen study (r10) for iridocyclitis. The AS data were procured from Cortes et al.’s GWAS [20], encompassing 9069 AS cases of European ancestry diagnosed based on modified New York criteria and 13,578 European ancestry controls. Iridocyclitis summary statistics were derived from the FinnGen study, comprising 8016 iridocyclitis cases and 390,647 controls, all involving European ancestry. Subtypes of iridocyclitis were delineated as acute or subacute (6755 cases) and chronic (1551 cases), with the same control group.

In the validation phase, GWAS data from FinnGen were employed for AS, encompassing 3162 cases and 294,770 controls, all involving European ancestry. For iridocyclitis, GWAS Catalog data comprised 134 cases diagnosed based on modified New York criteria and 456,214 controls, all involving European ancestry [21].

Subsequent experiments involved GWAS data from the FinnGen study (r10) for other HLA-B27 positive diseases. Reactive arthropathies included 3058 cases and 262,844 controls. Psoriatic arthropathies involved 3537 cases and 262,844 controls. Enteropathic arthropathies included 707 cases and 262,844 controls. All participants were of European ancestry.

### Mendelian randomization analysis

In this study, MR analysis was employed to establish causal relationships between plasma proteins and AS and between plasma proteins and iridocyclitis. We selected single-nucleotide polymorphisms (SNPs) that exhibited strong correlations with the exposures and reached genome-wide significance (P < 5 × 10^−8^) as instrumental variables, excluding SNPs from echo sequences. Initially, the instrumental strength of each SNP was determined using F statistics = (β_expose_/Se_expose_)^2^, and SNPs with F statistics > 10 were considered strong instrumental variables [22]. Then, we conducted tests for pleiotropy and heterogeneity on these SNPs and excluded those demonstrating pleiotropy or heterogeneity (P < 0.05) from subsequent analyses.

In the preliminary MR analysis, the choice of method depended on the availability of SNPs for each protein. The Wald ratio method was utilized when only one SNP was accessible for the protein; inverse variance weighting (IVW) was applied when two or more SNPs were accessible. To address multiple testing issues, we adjusted the results using the Bonferroni correction, with a significance threshold set at 0.05/(number of proteins used for analysis), prioritizing these outcomes for further investigation. The odds ratios (ORs) for an increased risk of disease are expressed as the per standard deviation (SD) increase in the plasma protein level.

For external validation of the proteins identified in the preliminary study, we employed a significance threshold of 0.05. After MR analysis, the Steiger Direction Test and Steiger Filtering were used to detect whether there was a reverse causal effect from disease to protein [23]. In cases in which the directionality of the MR results was ambiguous despite the aforementioned methods, we conducted reverse MR to validate the causal relationship between the plasma proteins and disease.

All MR analyses were performed using the R package ‘TwoSampleMR’ and visualized using the R packages ‘ggplot2’ and ‘ggVolcano’.

### Bayesian colocalization analysis

Bayesian colocalization analysis was employed to assess whether two traits share a common variant within a specific chromosomal region. This approach considers all SNPs within the region and offers valuable insights into the genetic factors influencing two traits, aspects not addressed by MR analysis. The analysis evaluated support for five distinct hypotheses: (1) the selected regions were not associated with either trait; (2) the selected regions were associated with trait 1 only; (3) the selected regions were associated with trait 2 only; (4) the selected regions were associated with both traits, but distinct causal variants influenced each trait; and (5) the selected regions were associated with both traits and shared a single causal variant for both traits [24]. Posterior probabilities for each hypothesis (H0, H1, H2, H3, and H4) were calculated. The analyses were conducted using default parameters. Colocalization between two traits in a specific region was considered robust if the posterior probability of shared causal variation (PH4) was ≥ 0.8.

Bayesian colocalization analysis was performed using the R package 'coloc'.

### Drug target identification

To assess the efficacy of the identified proteins, we searched the DrugBank database for identified target proteins associated with ankylosing spondylitis and iridocyclitis [25]. We then searched the identified proteins in the STRING database and filtered out the top 10 proteins that were most closely associated with them [26]. We subsequently included all the proteins obtained in the previous steps within the analysis utilized to construct the protein–protein interaction (PPI) network (minimum required interaction score = 0.4). The results of the PPI analysis were visualized utilizing Cytoscape (v3.9.1).

### Single-cell transcriptome analysis

#### Data download and quality control

The dataset used, GSE178833, was obtained from the Illumina NextSeq 500 platform on GPL18573, comprising of four aqueous humor samples of HLA-B27-positive anterior uveitis, two aqueous humour samples of HLA-B27-negative anterior uveitis, and one aqueous humour sample of infectious endophthalmitis [27]. All the patients in the dataset who were in the HLA-B27-positive group had spondyloarthritis.

Our analysis included samples from patients with HLA-B27-positive uveitis and HLA-B27-negative uveitis from the GSE178833 dataset. Cells meeting the following criteria were chosen for future analysis: (1) unique molecular identifier (UMI) > 500; (2) 200 < genes detected per cell < 2500; (3) percentage of mitochondrial genes < 20%; and (4) percentage of ribosomal genes > 5%; complexity [log_UMI_ (genes detected per cell)] > 0.8. Samples with poor data quality (‘b27po4’) were removed. In total, 10,081 cells were ultimately retained.

#### Dimensionality and clustering

The R package ‘Seurat’ was used for dimensionality and clustering analysis. Initially, the Log-Normalize and ScaleData algorithms were applied to the filtered data. The FindVariableFeature function was utilized to filter the identified genes for further analysis via principal component analysis (PCA); the percentage change between each principal component (PC) and the subsequent component was calculated. If the percentage was less than 5%, the current number of PCs (13) was selected for the next FindNeighbors function. The R package "Harmony" was used to remove batch effects between samples. The uniform manifold approximation and projection (UMAP) method was employed to conduct the analysis. The FindClusters function was used for cluster analysis. Using the ScType database, we identified specific cell type markers for immune cells, thereby determining the putative cell type for each cell population [28]. To ensure the reliability of the cell type annotations, we used an immune cell-specific marker to validate the results of automated annotations. The immune cell markers used were as follows: myeloid cells (CSF1R), monocytes (OLR1), macrophages (MARCO, F13A1), myeloid dendritic cells (FLT3, ZBTB46), T cells (IL7R, TRAC), CD8^+^ T cells (TRBC2, CD8A), CD4^+^ T cells (CD4), natural killer cells (NCR1, KLRF1, NCAM1), and B cells (CD19, MS4A1).

Afterwards, we investigated expression of target protein-encoding genes across different cell types and analysed differences between the HLA-B27-positive and HLA-B27-negative groups within each cell type. The results for dimensionality were visualized using the R package ‘plot1 cell’ [29]; other results were visualized using the R packages ‘Seurat’ and ‘ggplot2’.

### Transcription factor analysis

We used the R package ‘SCENIC’ to construct gene regulatory networks for different cell types within the HLA-B27-positive group [30]. We started by filtering genes, excluding those expressed in less than 1% of cells and those with low expression levels (those with less than 3 UMIs in 1% of cells). For subsequent analysis, we identified genes in the RcisTarget database and conducted GENIE3 analysis [31] to determine potential targets of each transcription factor based on coexpression data. We identified potential direct targets by conducting DNA-motif analysis using the R package ‘RcisTarget’. Finally, we analysed the network activity in individual cells and scored them using AUCell to determine their cellular status. The results of the analysis were analysed via the R packages ‘SCENIC’ and ‘ComplexHeatmap’.

### Pseudotime analysis

Pseudotime analysis was performed on myeloid cells using the R package ‘monocle2’ [32]. Initially, myeloid cell data were extracted from the Seurat object obtained in the preceding step, followed by redimensionality and clustering. Subsequently, these cell types were reannotated using the ‘Immune_All_High’ database within the R package ‘celltypist’ [33]. We also used markers for immune cells to ensure the accuracy of the automated annotation: monocytes (OLR1), macrophages (MARCO, F13A1), CD141^+^CLEC9A^+^ classic dendritic cells (cDCs) (IDO2, CLEC9A, THBD), monocyte-derived dendritic cells (moDCs) (CD1E, CD1C), and migratory dendritic cells (FSCN1, LAMP3, CCR7). The DCs were then categorized according to AFI1 expression status into high-AIF1 DCs (cDCs and moDCs) and low-AIF1 DCs (migratory dendritic cells).

We then transformed the Seurat object annotated into a monocle2 object. To present the hypothetical trajectory location of the cells, the orderCells function was employed to arrange them along a developmental axis. Finally, BEAM analysis was used to examine expression of genes responsible for determining cell fate at specific time points. Visualization of the aforementioned analysis was facilitated through utilization of R packages such as “monocle2”, “plot1cell”, “ggplot2”, and “ComplexHeatmap”.

### Cellular communication analysis

Intercellular communication analysis was conducted using the R package ‘CellChat’ [34]. The Seurat objects annotated were converted into CellChat objects and divided into groups based on HLA-B27 positivity or negativity. The function identifyOverExpressedGenes was used to determine which ligands or receptors are upregulated in each cell type; the function identifyOverExpressedInteractions was used to identify highly expressed pathways in each cell type. The projectData function was utilized to project gene expression data onto the PPI network. Finally, the netAnalysis_computeCentrality function was used to calculate the significance of the interaction. The above results were compared between groups and visualized using the R packages ‘CellChat’ and ‘ggplot2’.

### Role of the funding source

The funders did not have any role in the analysis or interpretation of the data, the writing of the manuscript, or the decision to submit the paper for publication.

## Results

### Screening for plasma risk proteins

#### MR analysis

Data for a total of 3233 SNPs of 1561 proteins were included in our preliminary analysis. First, we excluded 194 SNPs at levels that did not have genome-wide significance (P < 5 × 10^−8^). Prior to MR analysis, we excluded SNPs with pleiotropy and heterogeneity. Pleiotropy analysis did not reveal any instrumental variables associated with significant pleiotropy (P < 0.05). In heterogeneity analysis, we excluded 37 proteins for which the instrumental variables were heterogeneous (P < 0.05) (data not shown).

**Fig. 2:**
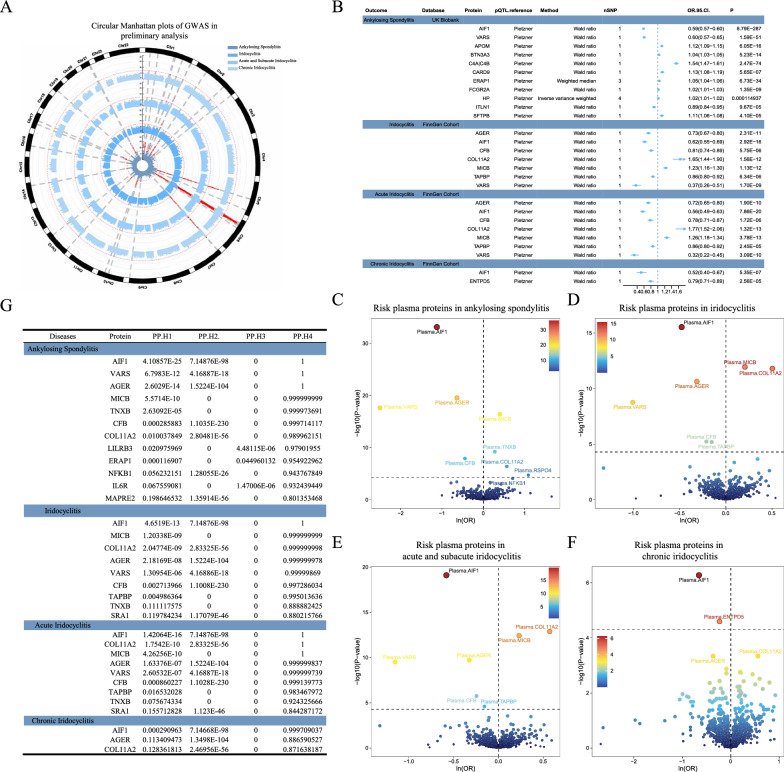
Preliminary MR analysis and colocalization analysis of pCAM proteins with AS, iridocyclitis and its subtypes. **A** Manhattan plot for AS, iridocyclitis and its subtypes. **B** Study of causal relationships between circulating proteins and AS, iridocyclitis and their subtypes using MR analysis. **C**–**F** Volcano plots of MR results for plasma proteins on the risk of AS (**C**), iridocyclitis (**D**), acute and subacute iridocyclitis (**E**) and chronic iridocyclitis (**F**). The horizontal black dotted line corresponds to the Bonferroni-corrected P value. **G** Bayesian colocalization results support the results of MR analysis. OR, increased risk of disease was expressed as a percentage of the SD increase in plasma protein levels; p value, proportion of variance explained. PPs H1-H4, posterior probabilities supporting the colocalization hypothesis

In preliminary MR analysis, we analysed the causal effects of plasma proteins on AS, iridocyclitis, acute and subacute iridocyclitis, and chronic iridocyclitis separately. Manhattan plots of the GWASs for the preliminary MR analysis are shown in Fig. 2A. We first used GWAS data for AS from UK Biobank and identified 11 proteins causally associated with AS by MR analysis (Fig. 2B, C), with increased plasma levels of three proteins, Allograft inflammatory factor 1(AIF-1) (OR = 0.59; 95% CI 0.57–0.60; P = 8.79 × 10^–287^), Valyl-tRNA synthetase (VARS) (OR = 0.60; 95% CI 0.57–0.65; P = 1.59 × 10^–51^), and Intelectin-1 (ITLN1) (OR = 0.89; 95% CI 0.84–0.95; P = 9.67 × 10^–5^), decreasing the risk of AS. The other eight proteins, Apolipoprotein M (APOM) (OR = 1.12; 95% CI 1.09–1.15; P = 6.06 × 10^–16^), Butyrophilin subfamily 3 member A3 (BTN3A3) (OR = 1.04; 95% CI 1.03–1.05; P = 5.23 × 10^–14^), Complement C4A (Rodgers blood group)|Complement C4B (Chido blood group)(C4A|C4B) (OR = 1.54; 95% CI 1.47–1.61; P = 2.47 × 10^–74^), Caspase recruitment domain family member 9 (CARD9) (OR = 1.13; 95% CI 1.08–1.19; P = 5.65 × 10^–7^), Endoplasmic reticulum aminopeptidase 1 (ERAP1) (OR = 1.05; 95% CI 1.04–1.06; P = 6.73 × 10^–34^), Fc gamma receptor Ila (FCGR2A) (OR = 1.02; 95% CI 1.01–1.03; P = 1.35 × 10^–9^), Haptoglobin (HP) (OR = 1.02; 95% CI 1.01–1.02; P = 1.15 × 10^–4^), and Surfactant protein B (SFTPB) (OR = 1.11; 95% CI 1.06–1.08; P = 4.10 × 10^–5^), increased the risk of AS.

We next explored the relationship between plasma proteins and iridocyclitis. We identified 7 proteins causally associated with iridocyclitis: AIF1 (OR = 0.62; 95% CI 0.55–0.69; P = 2.92 × 10^–16^), VARS (OR = 0.37; 95% CI 0.26–0.51; P = 1.70 × 10^–9^), AGER (OR = 0.73; 95% CI 0.67–0.80; P = 2.31 × 10^–11^), CFB(OR = 0.81; 95% CI 0.74–0.89; P = 5.75 × 10^–6^), COL11A2 (OR = 1.65; 95% CI 1.44–1.90; P = 1.56 × 10^–12^), MICB (OR = 1.23; 95% CI 1.16–1.30; P = 1.13 × 10^–12^) and TAPBP(OR = 0.86; 95% CI 0.80–0.92; P = 6.34 × 10^–6^) (Fig. 2B,D). In analysis of subtypes of iridocyclitis, we identified 7 proteins causally associated with acute and subacute iridocyclitis, including AIF-1 (OR = 0.56; 95% CI 0.49–0.63; P = 7.86 × 10^–20^), VARS (OR = 0.32; 95% CI 0.22–0.45; P = 3.09 × 10^–10^), AGER (OR = 0.72; 95% CI 0.65–0.80; P = 1.90 × 10^–10^), CFB (OR = 0.78; 95% CI 0.71–0.87; P = 1.72 × 10^–6^), COL11A2 (OR = 1.77; 95% CI 1.52–2.06; P = 1.32 × 10^–13^), MICB (OR = 1.26; 95% CI 1.18–1.34; P = 3.78 × 10^–13^), and TAPBP (OR = 0.86; 95% CI,0.80–0.92; P = 2.45 × 10^–5^) (Fig. 2B, E). Two proteins, AIF1 (OR = 0.52; 95% CI 0.40–0.67; P = 5.35 × 10^–7^) and ENTPD5 (OR = 0.79; 95% CI 0.71–0.89; P = 2.56 × 10^–5^), were causally associated with chronic iridocyclitis (Fig. 2B, F). Two proteins that are causally associated with AS and iridocyclitis are AIF1 and VARS, and AIF1 is causally associated with only acute and subacute iridocyclitis. The above instrumental variable information for plasma proteins is displayed in Additional file 1: Table S1.

We then verified that the causal relationship between plasma proteins and AS and iridocyclitis was correct by the Steiger test and Steiger filtering (Table 1). When using UKB data as the outcome data, the results showed that AS had reverse causality for VARS, AIF1 and C4A/C4B. The causal relationship between the remaining plasma proteins and disease was unidirectional. Reverse MR validation revealed that AS significantly reduced plasma VARS and AIF1 levels (Additional file 1: Table S2).

**Table 1:** Steiger test and Steiger filtering estimates for the causal direction between plasma proteins to AS and iridocyclitis

Protein	Outcome name	outcome reference	Steiger test correct causal direction	Steiger test pval	SNP	Steiger filtering correct causal direction	Steiger_pval
Plasma.AGER	Ankylosing spondylitis	FinnGen	TRUE	1.0997E−94	rs2070600	TRUE	1.3867E−90
Plasma.AIF1	Ankylosing spondylitis	FinnGen	TRUE	7.48619E−85	rs2261033	TRUE	1.84802E−78
Plasma.CFB	Ankylosing spondylitis	FinnGen	TRUE	1.8097E−183	rs641153	TRUE	8.8334E−210
Plasma.COL11A2	Ankylosing spondylitis	FinnGen	TRUE	1.03009E−54	rs3129205	TRUE	7.15834E−52
Plasma.TNXB	Ankylosing spondylitis	FinnGen	TRUE	6.8159E−182	rs45451301	TRUE	0
Plasma.VARS	Ankylosing spondylitis	FinnGen	TRUE	1.51682E−15	rs453821	TRUE	8.31624E−15
Plasma.AIF1	Ankylosing spondylitis	UK Biobank	FALSE	5.8074E−25	rs2261033	FALSE	5.21377E−24
Plasma.APOM	Ankylosing spondylitis	UK Biobank	TRUE	7.42183E−18	rs2255741	TRUE	1.96695E−17
Plasma.BTN3A3	Ankylosing spondylitis	UK Biobank	TRUE	1.20084E−58	rs9393711	TRUE	0
Plasma.C4A|C4B	Ankylosing spondylitis	UK Biobank	FALSE	0.002663452	rs3117580	FALSE	0.00444648
Plasma.CARD9	Ankylosing spondylitis	UK Biobank	TRUE	1.60674E−07	rs4077515	TRUE	3.84782E−08
Plasma.ERAP1	Ankylosing spondylitis	UK Biobank	TRUE	1.8175E−115	rs27895	TRUE	2.00675E−08
					rs467735	TRUE	0
					rs62364719	TRUE	5.50478E−85
Plasma.FCGR2A	Ankylosing spondylitis	UK Biobank	TRUE	3.41195E−66	rs4657041	TRUE	0
Plasma.HP	Ankylosing spondylitis	UK Biobank	TRUE	1.9314E−181	rs77303550	TRUE	5.8206E−282
Plasma.ITLN1	Ankylosing spondylitis	UK Biobank	TRUE	3.6041E−07	rs7532133	TRUE	1.03467E−06
Plasma.SFTPB	Ankylosing spondylitis	UK Biobank	TRUE	2.16399E−07	rs11126996	TRUE	7.75119E−07
Plasma.VARS	Ankylosing spondylitis	UK Biobank	FALSE	0.000107735	rs453821	FALSE	7.23814E−05
Plasma.AGER	Acute or subacute iridocyclitis	FinnGen	TRUE	3.183E−101	rs2070600	TRUE	5.84831E−97
Plasma.AIF1	Acute or subacute iridocyclitis	FinnGen	TRUE	1.95201E−92	rs2261033	TRUE	1.01221E−85
Plasma.COL11A2	Acute or subacute iridocyclitis	FinnGen	TRUE	1.05049E−52	rs3129205	TRUE	6.62665E−50
Plasma.VARS	Acute or subacute iridocyclitis	FinnGen	TRUE	8.05775E−18	rs453821	TRUE	5.08133E−17
Plasma.AIF1	Chronic iridocyclitis	FinnGen	TRUE	8.1108E−98	rs2261033	TRUE	6.64628E−91

#### Bayesian colocalization analysis

In our investigation, we applied Bayesian colocalization analysis to examine whether plasma proteins and AS and iridocyclitis share a common genetic variant within a specific chromosomal region (Fig. 2G).

The results of Bayesian colocalization analysis strongly support the hypothesis that 12 plasma proteins are linked to AS through the same genetic variant. These proteins include AGER (coloc.abf-PPH4 = 1.000), AIF1 (coloc.abf-PPH4 = 1.000), VARS (coloc.abf-PPH4 = 1.000), MICB (coloc.abf-PPH4 = 1.000), TNXB (coloc.abf-PPH4 = 1.000), CFB (coloc.abf-PPH4 = 1.000), COL11A2 (coloc.abf-PPH4 = 0.990), LILRB3 (coloc.abf-PPH4 = 0.979), ERAP1 (coloc.abf-PPH4 = 0.955), NFKB1 (coloc.abf-PPH4 = 0.943), IL6R (coloc.abf-PPH4 = 0.932), and MAPRE2 (coloc.abf-PPH4 = 0.8021).

Another 9 plasma proteins were identified to share the same genetic variant with iridocyclitis. These proteins include AIF1 (coloc.abf-PPH4 = 1.000), MICB (coloc.abf-PPH4 = 1.000), COL11A2 (coloc.abf-PPH4 = 1.000), AGER (coloc.abf-PPH4 = 1.000), VARS (coloc.abf-PPH4 = 1.000), CFB (coloc.abf-PPH4 = 0.997), TAPBP (coloc.abf-PPH4 = 0.995), TNXB (coloc.abf-PPH4 = 0.889), and SRA1 (coloc.abf-PPH4 = 0.880).

Furthermore, 9 plasma proteins were found to share the same genetic variant with acute and subacute iridocyclitis, which include AIF1 (coloc.abf-PPH4 = 1.000), COL11A2 (coloc.abf-PPH4 = 1.000), MICB (coloc.abf-PPH4 = 1.000), AGER (coloc.abf-PPH4 = 1.000), VARS (coloc.abf-PPH4 = 1.000), CFB (coloc.abf-PPH4 = 0.999), TAPBP (coloc.abf-PPH4 = 0.983), TNXB (coloc.abf-PPH4 = 0.924), and SRA1 (coloc.abf-PPH4 = 0.844).

Lastly, 3 plasma proteins share the same genetic variant with chronic iridocyclitis: AIF1 (coloc.abf-PPH4 = 1.000), AGER (coloc.abf-PPH4 = 0.887), and COL11A2 (coloc.abf-PPH4 = 0.872).

### Validation of target proteins in the plasma proteome

**Fig. 3:**
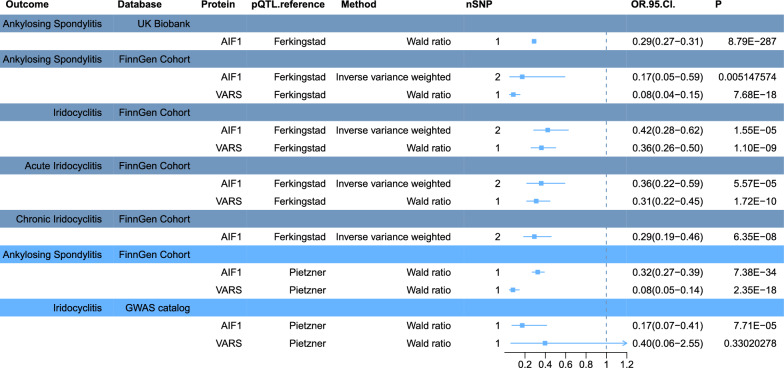
Causal relationships between two potential causal proteins and disease were externally validated. The pQTL data from Ferkingstad et al. were initially externally validated against disease GWAS data from a preliminary study (depicted in deep blue), followed by external validation against the pQTL data from a preliminary study using ankylosing spondylitis GWAS data from FinnGen and iridocyclitis GWAS data from GWAS Catalog (depicted in pale blue). OR, increased risk of disease was expressed as a per-SD increase in plasma protein levels

To validate our findings externally, we utilized pQTL data from Ferkingstad. Our MR analysis revealed significant associations between plasma AIF1 and VARS levels and the risk of developing AS. Specifically, both AIF1 and VARS levels were negatively associated with the risk of acute and subacute iridocyclitis development, and AIF1 levels were also negatively associated with the risk of developing chronic iridocyclitis (Fig. 3 (dark blue)). These associations were statistically significant (P < 0.05).

Furthermore, we conducted external validation using GWAS data on AS from the FinnGen study and GWAS data on iridocyclitis from GWAS Catalog. MR analysis confirmed the negative associations between plasma AIF1 and VARS levels and the risk of developing AS, as well as the negative association between AIF1 levels and the risk of developing iridocyclitis (Fig. 3 (pale blue)). These associations were statistically significant (P < 0.05).

### Exploring the causal relationship between target proteins and HLA-B27( +) disease

We employed Mendelian randomization (MR) analysis to investigate potential causal relationships between the risk proteins and other HLA-B27( +) diseases, as outlined in Table 2. Our analysis revealed a significant causal relationship between AIF1 and reactive arthropathies (OR = 0.66; 95% CI 0.55–0.80; P = 1.20 × 10^−5^).

**Table 2:** MR analysis to investigate potential causal relationships between risk proteins and other HLA-B27( +) diseases

Protein	Diseases	Diseases reference	Sample size	Method	Nsnp	OR (95% CI)	P(MR)	Steiger Test	P(ST)	Steiger Filtering	P(SF)
AIF1	Reactive arthropathies	FinnGen	243,675	Wald ratio	1	0.64(0.53–0.78)	5.21E−06	Passed	4.51E−95	Passed	2.64E−88
	Psoriatic arthropathies	FinnGen	244,048	Wald ratio	1	1.00(0.84–1.2)	0.992226967	Passed	1.15E−103	Passed	1.37E−96
	Enteropathic arthropathies	FinnGen	241,517	Wald ratio	1	0.68(0.46–1.01)	0.056096383	Passed	5.70E−100	Passed	5.01E−93
	Inflammatory bowel disease	FinnGen	377,277	Wald ratio	1	0.98(0.88–1.11)	0.788875671	Passed	1.0759E−104	Passed	1.56355E−97
VARS	Reactive arthropathies	FinnGen	243,675	Wald ratio	1	0.36(0.21–0.63)	0.000267246	Passed	2.14808E−18	Passed	1.38055E−17
	Psoriatic arthropathies	FinnGen	244,048	Wald ratio	1	0.57(0.35–0.92)	0.022049658	Passed	1.74869E−19	Passed	1.19174E−18
	Enteropathic arthropathies	FinnGen	241,517	Wald ratio	1	0.43(0.14–1.26)	0.121746744	Passed	4.45692E−20	Passed	3.13235E−19
	Inflammatory bowel disease	FinnGen	377,277	Wald ratio	1	1.47(1.07–2.02)	0.018367358	Passed	4.9002E−20	Passed	3.48487E−19

*MR* mendelian randomization, *CI* confidence interval, *ST* Steiger Test, *SF* Steiger Filtering

Additionally, we observed causal relationships between plasma VARS levels and several conditions, including reactive arthropathy (OR = 0.33; 95% CI 0.19–0.56; P = 4.00 × 10^−5^) and psoriatic arthropathy (OR = 0.52; 95% CI 0.32–0.84; P = 0.007).

To assess the directionality of these causal relationships, we conducted Steiger tests and Steiger filtering, which confirmed that these causal relationships are unidirectional.

### Exploring the drug target potential of recognized proteins

**Fig. 4:**
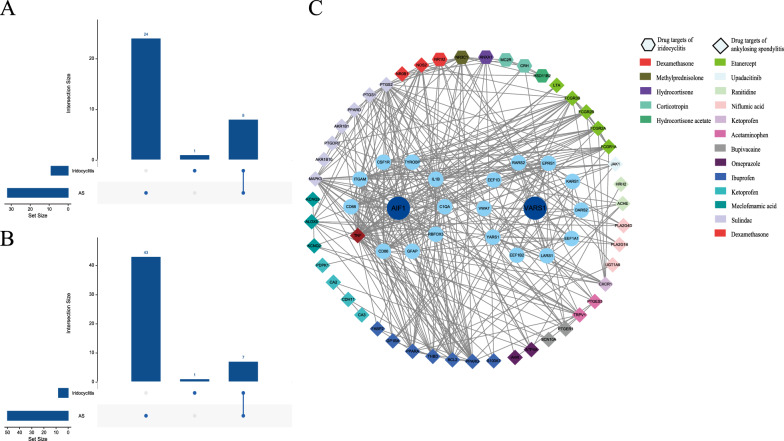
Interactions between current AS and iridocyclitis drug targets and recognized proteins. **A** Upset chart showing common drugs used to treat AS and iridocyclitis, with 8 drugs common to both diseases. **B** UpSet chart showing common drug targets between AS and iridocyclitis, with 7 drug targets common to both diseases. **C** PPI networks between drug targets and recognized proteins. Dark blue circle: recognized proteins; pale blue circle: the 10 proteins most closely related to recognized proteins according to the STRING database search; rhombic circle: drug targets of AS; hexagon: drug targets of iridocyclitis; red rhombus: hub protein. *PPI* protein‒protein interaction

We conducted a comprehensive search of the DrugBank database, focusing on drugs and drug targets associated with AS and iridocyclitis. The search yielded a total of 32 drugs utilized for AS and 9 drugs employed for iridocyclitis. Notably, 8 of these drugs were found to be shared between the two conditions (Fig. 4A). Our investigation of drug targets revealed 50 targets for AS and 8 targets for iridocyclitis, with 7 common targets for both diseases (Fig. 4B). Analysis of the PPI network included the mentioned protein targets, screened proteins, and the 10 proteins most closely related to the screened proteins. Consequently, the PPI network comprised 113 nodes and 351 PPI pairs (Fig. 4C). Among these proteins, tumour necrosis factor (TNF) exhibited close associations with both the screened proteins and the target proteins, suggesting its potential role as a hub protein.

### Identifying key cell types involved in the acute pathogenesis of HLA-B27-positive spondyloarthritis complicated with anterior uveitis

**Fig. 5:**
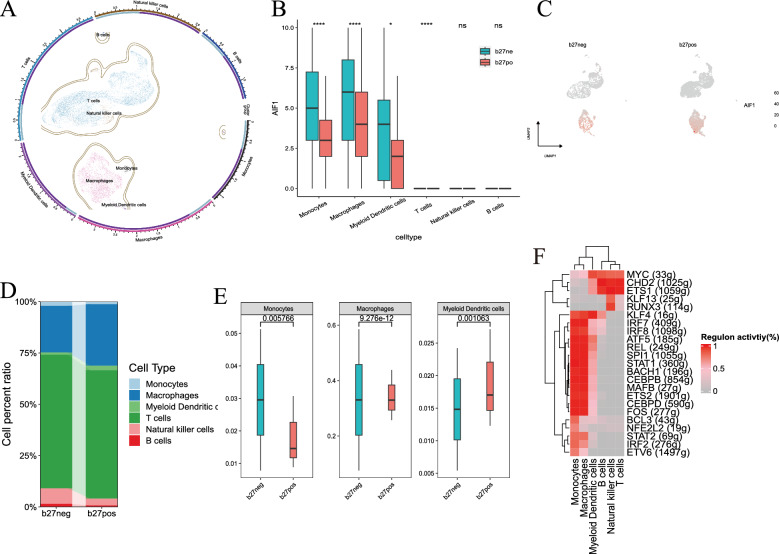
Identifying key cell types in the acute pathogenesis of HLA-B27 positive spondyloarthritis complicating anterior uveitis. **A** Clustering projection according to Seurat’s clustering system using UMAP as the dimension reduction method. **B** Box plots of expression levels of AIF1 across different cell types in both the HLA-B27-negative and HLA-B27-positive groups. *P < 0.05; ****P < 0.0001. **C** Dimensional reduction plot displaying the expression patterns of AIF1 in both the HLA-B27-negative and HLA-B27-positive groups. **D** Stacked chart of the proportions of different cell types within the HLA-B27-negative and HLA-B27-positive groups. **E** Box plots of the difference in the proportions of high AIF-expressing cell types between the HLA-B27-negative and HLA-B27-positive groups. **F** Heatmap of the activation of transcription factors across various cell types in the HLA-B27-positive group

Following stringent filtering, we retained a total of 10,081 cells from five samples for subsequent analysis (Additional file 1: Fig. S1). By referencing marker genes specific to each cluster, we assigned these clusters to 7 types of immune cells (Fig. 5A, Additional file 1: Fig. S1). Based on the clustering results, these cell types were broadly grouped into three main categories: Cluster 1 included monocytes, macrophages, and myeloid dendritic cells; Cluster 2 included CD4^+^ T cells, CD8^+^ T cells and natural killer cells; and Cluster 3 included naive B cells. Notably, VARS exhibited low expression across all cell types, and AIF1 demonstrated predominant expression within Cluster 1 (Fig. 5B and C). Interestingly, AIF1 expression in each of the Cluster 1 cells was significantly lower in the HLA-B27-positive group than in the HLA-B27-negative group (Fig. 5B). Furthermore, we observed a greater overall proportion of Cluster 1 cells in the HLA-B27-positive subgroup (Fig. 5D), with macrophages and myeloid dendritic cells being significantly more prevalent in the HLA-B27-positive subgroup than in the HLA-B27-negative subgroup (Fig. 5E).

Subsequently, we applied the SCENIC AUCell algorithm to cells in the HLA-B27-positive group. This analysis revealed a total of 23 regulons active in the HLA-B27-positive group. Notably, activation of transcription factors in Cluster 1 and other cell clusters exhibited significant disparities (Fig. 5F).

### Exploring the differentiation trajectory of high AIF-expressing cell types

**Fig. 6:**
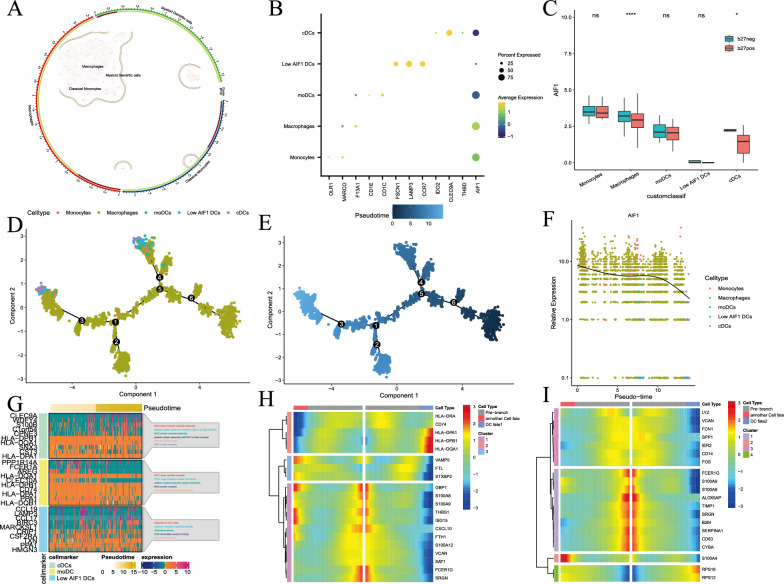
Exploring the differentiation trajectory of high-AIF-expressing cell types. **A** UMAP was utilized as a dimensionality reduction technique to project a reclustered Seurat clustering system of high-AIF-expressing cell types. **B** Bubble plot of myeloid cell subset marker genes. **C** Box plot of expression patterns of AIF1 in the HLA-B27-negative and HLA-B27-positive groups. **D**, **E** Pseudotime trajectory analysis showing high AIF-expressing cell types. The cells are coloured according to cell type (**D**) and pseudotime value (**E**). **F** The trend of AIF1 expression along pseudotime variation. **G** Heatmap of expression of the top 5 marker genes for three distinct subsets of dendritic cells, along with enrichment analysis. **H** BEAM analysis of predifferentiated nodes associated with DC fate 1. **I** BEAM analysis of predifferentiated nodes according to DC fate 2. BEAM, branched expression analysis modelling

To elucidate the precise mechanism underlying the acute onset of SpA/HLA-B27-associated AAU, we focused on cells with high AIF expression and conducted detailed analysis. Specifically, we isolated Cluster 1 cells and subjected them to repeated dimensionality reduction and clustering analysis. As a result, we successfully identified and annotated 5 distinct cell subtypes (Fig. 6A). Expression of marker genes in these cells is shown in a bubble plot in Fig. 6B.

Additionally, our examination of AIF1 expression yielded intriguing findings. Notably, AIF1 expression was lower in monocytes and in cDCs in the HLA-B27-positive group than in those in the HLA-B27-negative group (Fig. 6C).

Furthermore, trajectory analysis revealed interesting insights into cell type transitions. DCs were positioned at the ends of the branches (Fig. 6D, E). Notably, AIF1 expression decreased progressively during cell differentiation (Fig. 6F). Further analysis revealed enrichment of the initial 10 DC marker genes, indicating significant enrichment of MHC-II-related pathways in both the cDC and moDC subtypes and significant enrichment of migration-related pathways in the low-AIF1 DC population (Fig. 6G).

Finally, to further explore the underlying genetic factors, we conducted BEAM analysis focusing on pre-DC cell differentiation nodes. This analysis highlighted the top 20 genes displaying the most significant differences before and after the cell differentiation node (Fig. 6H, I).

### Inferencing intercellular interactions

**Fig. 7:**
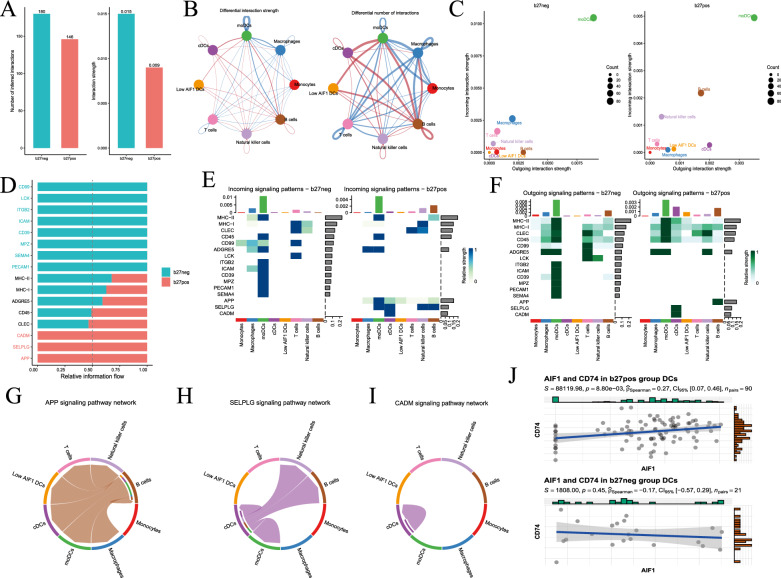
Analysis of intercellular communication among various cell types in the HLA-B27-negative and -positive groups. **A** Bar graph illustrating the quantity and strength of cell communication between the HLA-B27-negative and HLA-B27-positive groups. **B** Chord plot of the quantity and strength of cell communication among different cell types in the HLA-B27-negative and HLA-B27-positive groups. **C** Scatter plot representing the relative strength of cell communication for each cell type within the respective groups. **D** Specific cell communication pathways unique to the HLA-B27-negative and -positive groups. **E** The incoming strength of intergroup-specific pathways within various cell types. **F** The outcoming strength of intergroup-specific pathways within various cell types. **G**–**I** Presentation of specific pathways (the APP pathway, the SELPLG pathway and the CADM pathway) exclusive to the HLA-B27-positive group, depicting the direction of cell communication indicated by arrows. **J** The correlation between expression of AIF1 and that of the receptor for the APP pathway (CD74)

In the final phase of our analysis, we utilized CellChat to deduce intercellular communication patterns among various cell types within the two cell groups. In the HLA-B27-positive group, we identified a total of 180 cellular communication pathways; 146 pathways in the HLA-B27-negative group were revealed. The HLA-B27-positive group had fewer and generally weaker cellular connections (Fig. 7A). Specifically, compared to their counterparts in the HLA-B27-negative group, cDCs and low-AIF1 DCs in Cluster 1 of the HLA-B27-positive group exhibited more robust and frequent communication with other cells (Fig. 7B).

Further analysis involved comparing the relative strength of cellular communication across various cell types between the HLA-B27-positive and -negative groups. Notably, the outgoing interaction strength of cDCs was comparatively enhanced. Conversely, moDCs and low-AIF1 DCs exhibited a reduction in incoming interaction strength, with no significant change in outgoing interaction strength (Fig. 7C).

Subsequently, we identified group-specific cellular communication pathways, singling out the APP, SELPLG and CADM pathways as unique communication pathways within the HLA-B27-positive group (Fig. 7D). Remarkably, cDCs displayed the most significant difference in cellular communication strength between the HLA-positive and -negative groups (Fig. 7 E, F). The APP pathway receptor CD74 was found in all cell types, with its ligand (APP) expressed in B cells (Fig. 7G). Ligands for the SELPLG pathway (cDCs) were primarily expressed in cDCs, with their receptor (SELL) found in moDCs, cDCs, natural killer cells and B cells (Fig. 7H). Ligands (CADM1) and receptors (CADM1) for the CADM pathway were primarily expressed in cDCs (Fig. 7I).

Finally, we examined the correlation between AIF1 and these three key pathway receptors. Notably, AIF1 in the HLA-B27-positive group exhibited a correlation with the APP pathway receptor (CD74), which was more pronounced than that in the HLA-B27-negative group (Fig. 7J).

## Discussion

In this study, we employed a combination of Mendelian randomization (MR) and single-cell transcriptome analysis to comprehensively examine the acute pathogenesis of SpA/HLA-B27-associated anterior uveitis. Circulating AIF1 levels were shown to reduce the risk of SpA/HLA-B27-associated AAU. AIF1 is predominantly expressed by myeloid cells. The decrease in plasma AIF1 levels in SpA/HLA-B27-associated AAU patients is attributed to two factors: reduced AIF1 expression in myeloid cells and differentiation of monocyte-macrophages into DCs.

AIF1, initially discovered and cloned from rat heart grafts with chronic cardiac rejection, is a cytokine-responsive molecule in macrophages [35]. AIF1 expression is modulated by various inflammatory stimuli, including IFN-γ, TNF-α, interleukin-1β (IL-1β), and T-cell conditioned media [36]. The AIF1 gene is encoded within the major histocompatibility complex (MHC) class III region of chromosome 6p21.33 [37]. AIF1 expression is modulated by various inflammatory stimuli, including IFN-γ, TNF-α, interleukin-1β (IL-1β), and T-cell conditioned media [38]. Previous studies have linked AIF1 to various autoimmune diseases, including anti-GBM nephritis [39], rheumatoid arthritis [40], autoimmune rat nervous system lesions [41], and cardiac allograft rejection [42]. Under these conditions, AIF1 exacerbates inflammatory responses. However, prior to our study, no association had been established between AIF1 and SpA/HLA-B27-associated AAU. Thus, for the first time, our research unveiled a connection between AIF1 and SpA/HLA-B27-associated AAU.

Our investigation categorized DCs into three subgroups: CD141^+^ CLEC9A^+^ classic dendritic cells (cDCs), monocyte-derived dendritic cells (moDCs), and migratory dendritic cells. Furthermore, based on expression of AIF1, we delineated these cells into AIF1-high-expressing DCs (cDCs and moDCs) and AIF1-low-expressing DCs (migratory dendritic cells). The findings also highlighted the critical roles of AIF1 and dendritic cells (DCs) in the pathogenesis of SpA/HLA-B27-associated AAU. According to our pseudotime analysis, expression of AIF1 decreased with increasing pseudotime. Simultaneously, DCs are distributed at the terminal branches of the myeloid cell differentiation pathway. AIF1 may be associated with differentiation of myeloid cells, facilitating a greater propensity for differentiation towards dendritic cells within the myeloid lineage. This can simultaneously account for the decrease in the proportion of monocytes/macrophages and the increase in the proportion of dendritic cells. Cell communication analysis revealed cDCs play a pivotal role in cellular communication processes within the Spa/HLA-B27 group. Among them, the APP pathway, SELPLG pathway, and CADM pathway are distinctive pathways specific to the Spa/HLA-B27 group and are associated with cDCs. Additionally, expression of the receptor CD74 in the APP pathway correlated with that of AIF1 in cDCs.

Similarly, other researchers have shown that expression of AIF1 in DCs may correlate with Spa/HLA-B27-associated AAU. Silencing AIF1 in DCs has been shown to impede differentiation of CD4^+^ T cells into T helper cells that produce IL-17 and IFN-γ, two cytokines linked to AAU pathogenesis [43]. These two cytokines have been shown to be associated with AAU pathogenesis [44].

Other studies have proposed AIF1 as a significant player in the pathogenesis of SpA/HLA-B27-associated AAU. (1) AIF1 and microglia: AIF1 is widely expressed in microglia [45, 46]. Early studies identified AIF1 as a marker of microglial activation [47], but later, it was found that AIF1 could not be used to distinguish functional microglial phenotypes [48]. Keren-Shaul et al. identified a new subtype of microglia, rodent disease-associated microglia (DAMs), by transcriptional single-cell sorting; these cells have downregulated expression of AIF1 and several homeostatic genes [49]. This may suggest that AIF1 plays a role in maintaining homeostasis in the immune environment. (2) AIF1 and FOXP3^+^ macrophages: recent studies have shown that AIF1 and FOXP3 colocalize in macrophages and that AIF^+^/FOXP3^+^ cells can inhibit neural inflammation [50]. FOXP3 is an immunosuppressive transcription factor for regulatory T cells (Tregs) that suppresses sterile and pathogen-induced inflammation [51]. Whether AIF1 and FOXP3 play synergistic roles is worth exploring.

However, these cells primarily function within tissues, raising the question of how to account for the decrease in AIF1 levels in plasma. Indeed, the reason for the decrease in plasma AIF1 levels remains unclear. Plasma proteins originate from either active cellular secretion or passive release following cell death. To date, no study has conclusively identified the source of AIF1 in plasma. Nevertheless, many studies have shown that AIF1 can be actively secreted by myeloid immune cells [52–54]. Two hypotheses can be proposed based on the source of AIF1 in plasma. Hypothesis 1: Plasma AIF1 originates from the passive release of monocyte macrophages into the bloodstream upon cell death. Consequently, reduced plasma AIF1 levels may indicate the migration of monocyte macrophages into tissues and their subsequent differentiation into dendritic cells (DCs). Due to the convenience of detecting plasma proteins, plasma AIF1 levels might serve as a reliable predictor of SpA/HLA-B27-associated AAU development. Hypothesis 2: AIF1 in plasma results from active secretion by myeloid cells in the bloodstream. This scenario suggests the existence of an undiscovered regulatory network downstream of plasma AIF1 in the pathogenesis of SpA/HLA-B27-associated AAU. AIF1 may be a potential predictive marker and a promising drug target.

Another key plasma protein identified in our study, VARS, is an aminoacyl-tRNA synthase categorized as an anti-aminoacyl-tRNA synthetase (anti-ARS) [55]. Limited research has explored this molecule, with only one study identifying VARS as playing a pivotal role in nervous system development. Although the direct link between VARS and this disease has not been determined, anti-aminoacyl-tRNA synthetase (anti-ARS) antibodies have been unequivocally implicated as causative agents of anti-ARS synthetase syndrome [56], which is characterized by clinical manifestations such as interstitial lung disease, arthritis, and myositis [57, 58]. In this syndrome, eight anti-ARS antibodies have been identified, potentially correlating with clinical features [57]. Notably, autoantibodies targeting the remaining four ARSs (CysARS, ValARS, SerARS, and TrpARS) have not been identified. Notably, anti-VARS antibodies were found in a 2022 study in a patient with anti-ARS synthetase who had neither ILD nor myositis [59]. Our study identified low VARS levels in plasma as a risk factor for HLA-B27-associated AAU. Whether this low VARS level is due to an anti-ARS synthetase is a question worth investigating.

Further research is needed to understand the pathogenesis of Spa/HLA-B27-associated AAU. The gut microbiota may play a role in the development of acute conditions by affecting immune cells, but there is no direct evidence linking gut microbiota to AIF1 expression in DCs. The study is based on publicly available GWAS summary data, which lacks detailed information such as gender for stratified analysis. However, previous research suggests a higher prevalence of males and bilateral involvement [60]. Currently, there are no cellular or animal models available to study Spa/HLA-B27-associated AAU. Therefore, research based on extensive data and multiomics approaches is required. This study employed cross-validation with data from diverse sources to ensure the stability and accuracy of research findings.

In summary, our study revealed that differentiation of myeloid cells into DCs and downregulation of AIF1 within cDCs play pivotal roles in the acute pathogenesis of SpA/HLA-B27-associated anterior uveitis. Furthermore, we established that plasma AIF1 levels can serve as a reliable predictor of SpA/HLA-B27-associated AAU and suggest the potential of plasma AIF1 as a promising drug target in this context.

### Supplementary Information

**Additional file 1.** Instrumental variable information for key plasma proteins

**Additional file 2.** Inverse MR results for AS and key plasma proteins

**Additional file 3.** Cellular quality control for single-cell data

**Additional file 4.** Bubble plot of marker genes

## Data Availability

All the data used in this study are available in the public repository.
