# Supplementary figures and images for: Investigation of the acute pathogenesis of spondyloarthritis/HLA-B27-associated anterior uveitis based on genome-wide association analysis and single-cell transcriptomics
Source: J Transl Med. 2024 Mar 12;22:271. doi: 10.1186/s12967-024-05077-y (PMC10936029; doi:10.1186/s12967-024-05077-y)

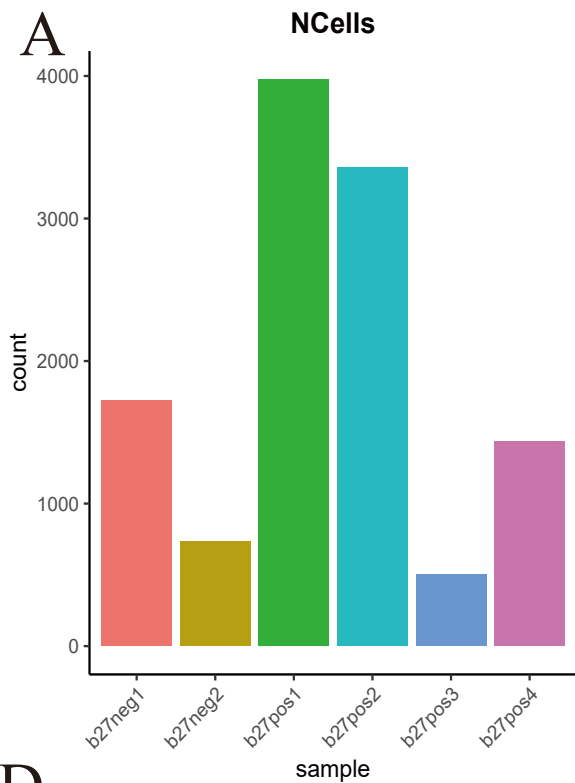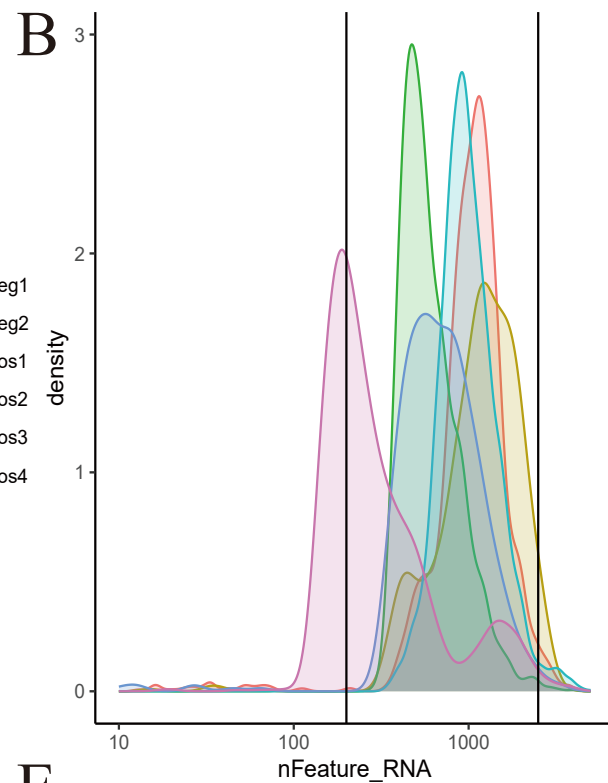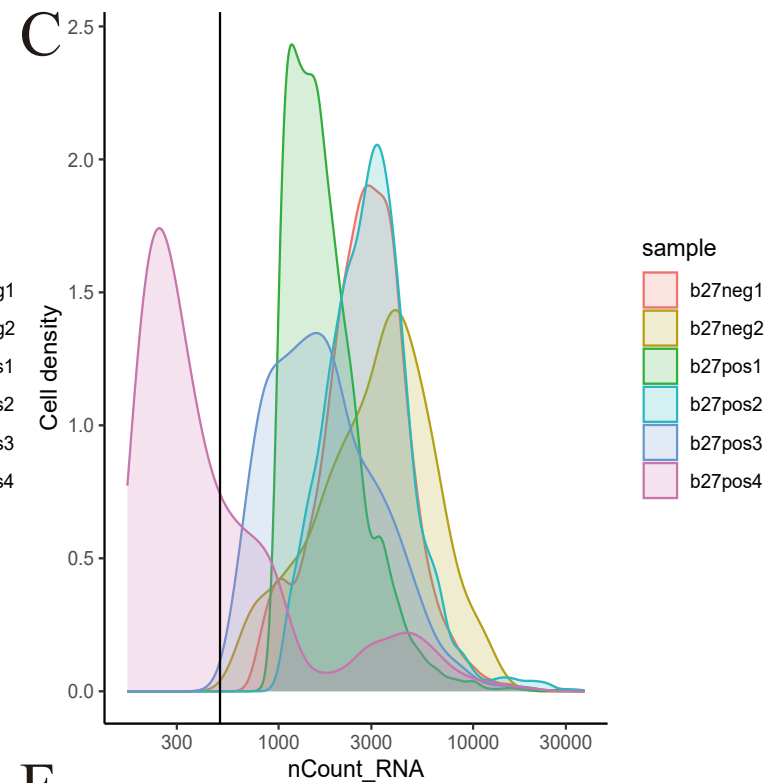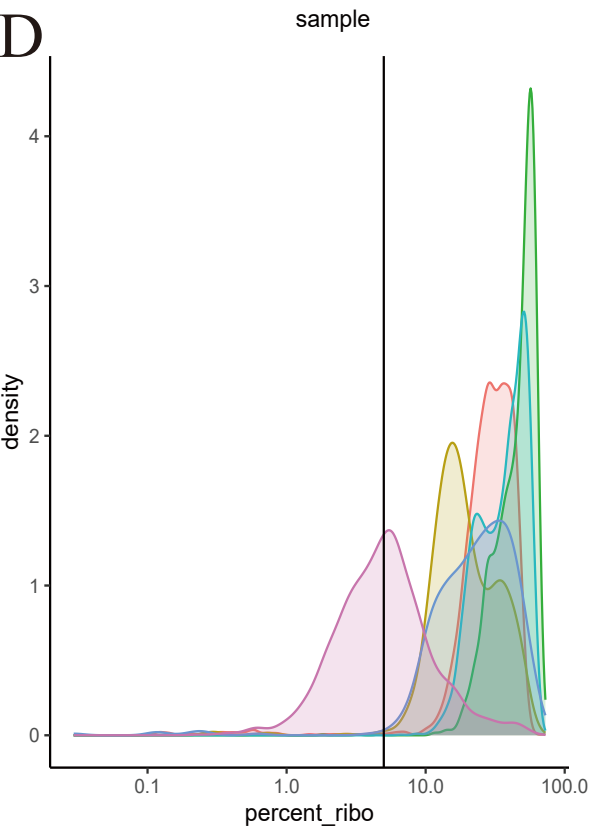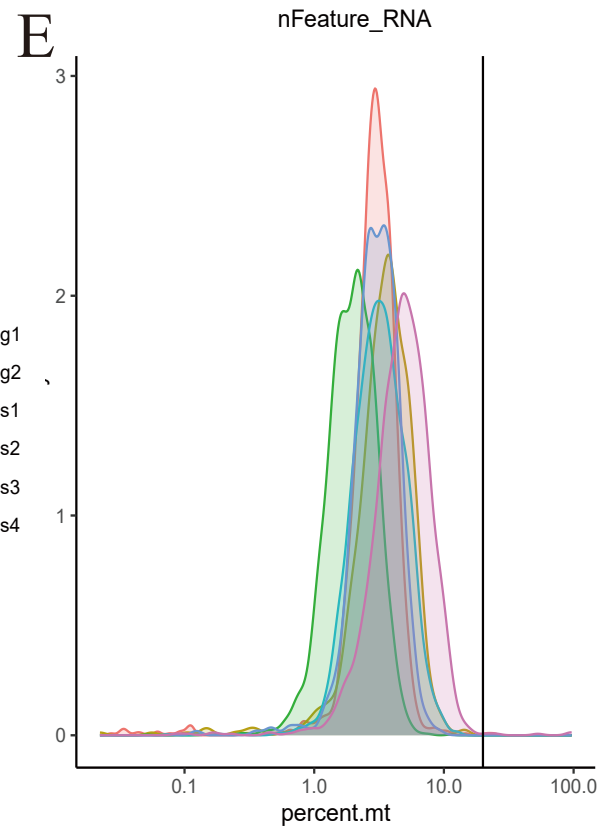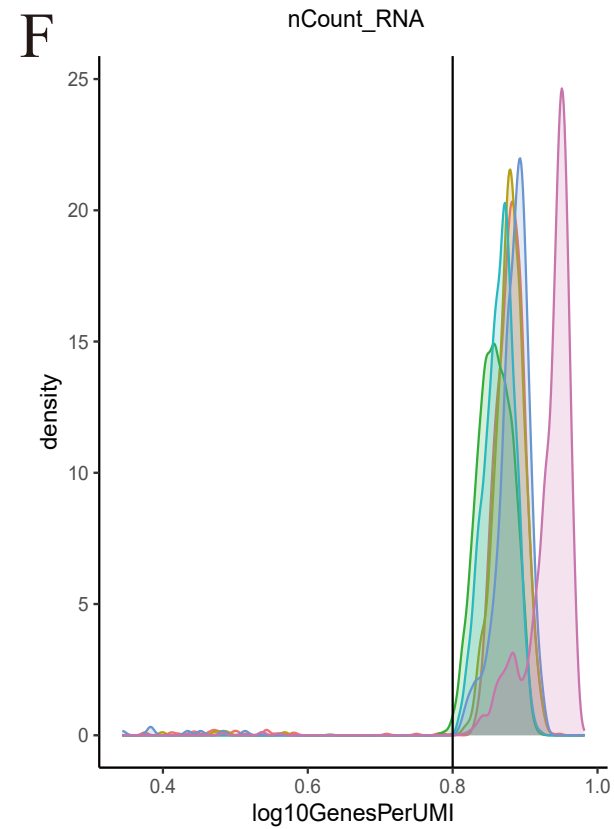

Supplement: Supplementary file 3 — Additional file 3. Cellular quality control for single-cell data [file 12967_2024_5077_MOESM3_ESM.pdf]

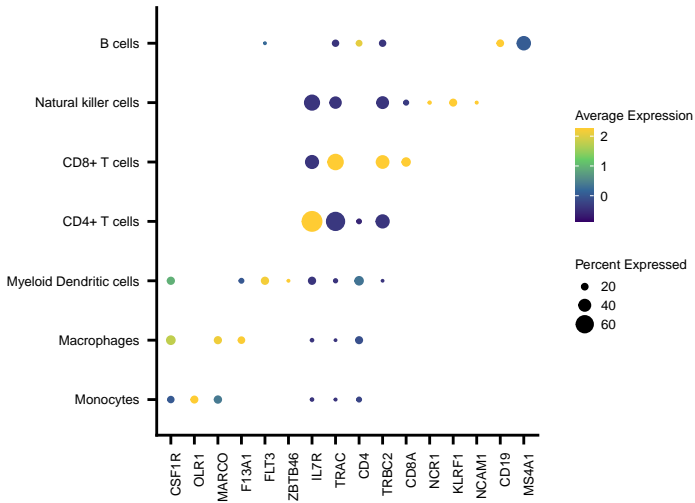

Supplement: Supplementary file 4 — Additional file 4. Bubble plot of marker genes [file 12967_2024_5077_MOESM4_ESM.pdf]
